# Disconnection between the default mode network and medial temporal lobes in post-traumatic amnesia

**DOI:** 10.1093/brain/aww241

**Published:** 2016-10-22

**Authors:** Sara De Simoni, Patrick J. Grover, Peter O. Jenkins, Lesley Honeyfield, Rebecca A. Quest, Ewan Ross, Gregory Scott, Mark H. Wilson, Paulina Majewska, Adam D. Waldman, Maneesh C. Patel, David J. Sharp

**Affiliations:** aww241-1 1 Computational, Cognitive and Clinical Neuroimaging Laboratory, Imperial College London, Division of Brain Sciences, Hammersmith Hospital, London, UK; aww241-2 2 Department of Imaging, Charing Cross Hospital, London, UK; aww241-3 3 Traumatic Brain Injury Centre, Imperial College, St Mary’s Hospital, London, UK

**Keywords:** post-traumatic amnesia, traumatic brain injury, functional connectivity, default mode network, memory

## Abstract

**See Bigler (doi:10.1093/aww277) for a scientific commentary on this article**.

Post-traumatic amnesia is very common immediately after traumatic brain injury. It is characterized by a confused, agitated state and a pronounced inability to encode new memories and sustain attention. Clinically, post-traumatic amnesia is an important predictor of functional outcome. However, despite its prevalence and functional importance, the pathophysiology of post-traumatic amnesia is not understood. Memory processing relies on limbic structures such as the hippocampus, parahippocampus and parts of the cingulate cortex. These structures are connected within an intrinsic connectivity network, the default mode network. Interactions within the default mode network can be assessed using resting state functional magnetic resonance imaging, which can be acquired in confused patients unable to perform tasks in the scanner. Here we used this approach to test the hypothesis that the mnemonic symptoms of post-traumatic amnesia are caused by functional disconnection within the default mode network. We assessed whether the hippocampus and parahippocampus showed evidence of transient disconnection from cortical brain regions involved in memory processing. Nineteen patients with traumatic brain injury were classified into post-traumatic amnesia and traumatic brain injury control groups, based on their performance on a paired associates learning task. Cognitive function was also assessed with a detailed neuropsychological test battery. Functional interactions between brain regions were investigated using resting-state functional magnetic resonance imaging. Together with impairments in associative memory, patients in post-traumatic amnesia demonstrated impairments in information processing speed and spatial working memory. Patients in post-traumatic amnesia showed abnormal functional connectivity between the parahippocampal gyrus and posterior cingulate cortex. The strength of this functional connection correlated with both associative memory and information processing speed and normalized when these functions improved. We have previously shown abnormally high posterior cingulate cortex connectivity in the chronic phase after traumatic brain injury, and this abnormality was also observed in patients with post-traumatic amnesia. Patients with post-traumatic amnesia showed evidence of widespread traumatic axonal injury measured using diffusion magnetic resonance imaging. This change was more marked within the cingulum bundle, the tract connecting the parahippocampal gyrus to the posterior cingulate cortex. These findings provide novel insights into the pathophysiology of post-traumatic amnesia and evidence that memory impairment acutely after traumatic brain injury results from altered parahippocampal functional connectivity, perhaps secondary to the effects of axonal injury on white matter tracts connecting limbic structures involved in memory processing.

## Teaser

Post-traumatic amnesia is common following traumatic brain injury and is an important predictor of clinical outcome. De Simoni *et al.* report that post-traumatic amnesia results from a temporary disruption to functional brain networks involved in memory processing, with functional connectivity between the parahippocampal gyrus and posterior cingulate cortex particularly affected.

## Introduction

Post-traumatic amnesia (PTA) frequently follows traumatic brain injury (TBI) and is characterized by transient anterograde amnesia, confusion, disorientation and agitation (Marshman *et al.*, 2013). Together with attentional deficits, the inability to encode new memories is at the core of the syndrome, which has a highly variable duration lasting between seconds and months (Ahmed *et al.*, 2000). The length of PTA is an important index of injury severity and predicts functional outcome (Walker *et al.*, 2010; Konigs *et al.*, 2012; Eastvold *et al.*, 2013). However, despite its prevalence and clinical importance, there is still no clear understanding of its pathophysiological basis.

Previous studies have shown that PTA is sometimes associated with focal lesions and decreased cerebral perfusion, mainly in the frontal and temporal lobes (Lorberboym *et al.*, 2002; Gowda *et al.*, 2006; Metting *et al.*, 2010). Some studies have reported that the extent of perfusion changes predicts the severity of PTA (Lorberboym *et al.*, 2002; Metting *et al.*, 2010). However, PTA can be seen in patients without focal lesions and can also occur in cases of mild TBI in the absence of any overt structural abnormalities (Metting *et al.*, 2010). Indeed, the vast majority of patients with short duration PTA have no evidence of focal injuries. The lack of a clear relationship with obvious structural injury and its transient nature suggests that PTA results from a temporary disruption in the interactions of brain regions involved in memory processing.

The hippocampus and parahippocampus are critical for memory (Scoville and Milner, 1957; Schachter and Wagner, 1999; Cabeza and Nyberg, 2000). These medial temporal lobe (MTL) structures interact with widespread cortical regions to support encoding, consolidation and retrieval processes during memory (Girardeau and Zugaro, 2011; Logothetis *et al.*, 2012; Poch and Campo, 2012). Electrophysiological studies have shown that hippocampal theta oscillatory activity is present during encoding conditions. In subsequent ‘off-line’ memory consolidation the hippocampus exhibits a pattern of activity characterized by sharp wave-ripple (SPWR) complexes, proposed to allow information transfer between hippocampal and neocortical areas. Its suppression disrupts memory consolidation (Girardeau and Zugaro, 2011). Recently, a combined electrophysiological-functional MRI study of memory consolidation in non-human primates demonstrated that hippocampal ripples during ‘rest’ or sleep are associated with activation of widespread cortical areas. Hippocampal activity was particularly correlated with that of the posterior cingulate cortex (PCC) and retrosplenial cortex (Logothetis *et al.*, 2012).

In humans, interactions between the MTL and other cortical areas can be studied by investigating activity within intrinsic connectivity networks, defined using functional MRI. A number of limbic structures involved in memory processing are linked within one particular network, the default mode network (DMN) (Raichle *et al.*, 2001; Vincent *et al.*, 2006; Smith *et al.*, 2009). The DMN consists of cortical brain regions including the posterior cingulate and retrosplenial cortices, precuneus, lateral inferior parietal lobes, inferior temporal gyri and ventromedial prefrontal cortex (vmPFC). The hippocampus and parahippocampus belong to an MTL subsystem of the DMN that dynamically interfaces with the rest of the network (Andrews-Hanna *et al.*, 2014). The parahippocampus appears to play a central role in mediating the functional connection between this MTL subsystem and the rest of the DMN, particularly in the absence of external stimulation (Huijbers *et al.*, 2011; Ward *et al.*, 2014). The DMN is active at rest and during episodic memory retrieval tasks, and shows decreased activity during tasks that require external allocation of attention (Raichle *et al.*, 2001; Greicius *et al.*, 2003). As a key node of the DMN, PCC activation in particular has been implicated in successful episodic retrieval (Daselaar *et al.*, 2009; Kim *et al.*, 2010).

The strength of interactions (functional connectivity) within the DMN is important for successful memory formation. For example, interactions between two nodes of the DMN, the PCC and vmPFC, influence associative and working memory function (Hampson *et al.*, 2006; Andrews-Hanna *et al.*, 2007). Connectivity between the PCC/precuneus and MTL during resting state conditions is also predictive of associative memory performance in healthy controls, and disruptions to these connections are found in a variety of disorders where memory is impaired, including Alzheimer’s disease and amnestic mild cognitive impairment (Wang *et al.*, 2006, 2010; Zhou *et al.*, 2008; Dunn *et al.*, 2014).

In the context of TBI, functional network abnormalities are linked to underlying diffuse axonal injury, which appears to disrupt communication within and between brain networks (Sharp *et al.*, 2011; Bonnelle *et al.*, 2012; Jilka *et al.*, 2014). Hence, a functional disconnection between the MTL subsystem and the rest of the DMN could result from structural damage within white matter tracts that connect them. One candidate white matter tract is the cingulum bundle. This projects from the PCC to both the vmPFC and parts of the MTL, in particular the parahippocampal gyrus (Schmahmann *et al.*, 2007; Jones *et al.*, 2013). In addition, the PCC also has connections that terminate in the precuneus, parietal lobes, retrosplenial cortex, and entorhinal cortex, which itself has direct connections to the hippocampus (Parvizi *et al.*, 2006). Persistent memory impairments after TBI are associated with damage to the connections of the hippocampus. In particular, the structural integrity of the fornix is correlated with the extent of associative memory impairment (Kinnunen *et al.*, 2011).

Taken together these studies motivate an investigation of whether PTA-associated amnestic symptoms result from a functional and/or structural disconnection between MTL brain regions and the PCC as a key node of the DMN. For the first time, we use advanced brain imaging techniques, including functional MRI and diffusion tensor imaging (DTI), to study the pathophysiological basis of PTA. Previous studies have questioned whether PTA is a predominantly mnemonic or attentional disorder (Stuss *et al.*, 1999; Tittle and Burgess, 2011). This study focuses primarily on identifying the neural correlates of PTA-associated mnemonic deficits. We test a number of specific hypotheses: (i) PTA is associated with a functional disruption within the DMN. Based on our previous work we expected to see an increase in functional connectivity within posterior nodes of the DMN following TBI (Sharp *et al.*, 2011); (ii) PTA is associated with a disruption of functional connectivity between the PCC and MTL structures (the hippocampus and the parahippocampus), which normalizes following the resolution of PTA; and (iii) PTA is associated with diffuse axonal injury to the cingulum bundle.

## Materials and methods

### Participant demographics and clinical details

#### Patient group

Nineteen patients with a recent history of TBI were recruited from the Major Trauma Ward, St Mary’s Hospital, London, UK (Supplementary Table 1). Patients were included in the study if they were between the ages of 16 and 80, had no significant premorbid psychiatric or neurological history, alcohol or substance misuse, significant previous TBI, and were clinically stable. Exclusion criteria included significant language or visuospatial impairments, contraindication to MRI, inability to tolerate the scanner environment and neurosurgery. According to the Mayo Classification (Malec *et al.*, 2007), all patients recruited were classified as moderate-severe. Patients were scanned in the afternoon after completion of the neuropsychological testing. Written informed consent was obtained from all patients judged to have capacity by a trained clinician. Patients in PTA who were judged not to have capacity were deemed unable to give informed consent for participation in the study. This issue was addressed by obtaining written assent from these patients at the acute stage as well as informed written assent on behalf of the patient from a caregiver. Retrospective consent was obtained for all these patients when they emerged from PTA. Written informed consent was obtained from all patients judged to have capacity according to the Declaration of Helsinki. No patients withdrew their consent once they had emerged from PTA. The study was approved by the West London Research Ethics Committee (09/H0707/82).

**Figure 1:**
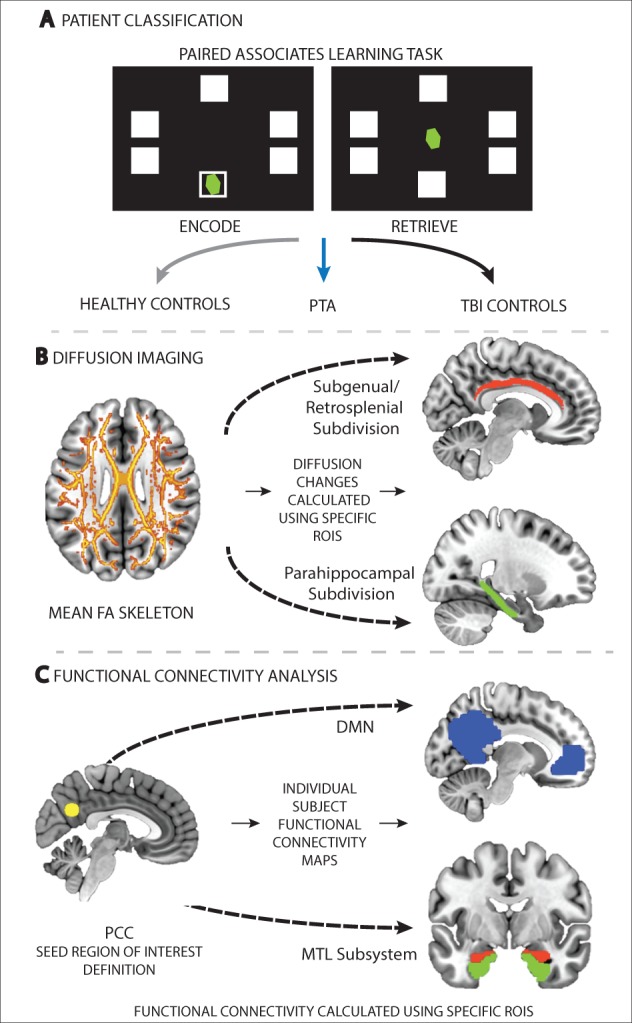
**Overview of the neuropsychological and imaging methods used to assess (A) memory performance, (B) white matter structural integrity, and (C) functional connectivity in both TBI patients and controls.** (**A**) Patients were classified into the PTA or traumatic brain injury control (TBIC) groups depending on their performance on the PAL task. (**B**) White matter integrity was investigated using DTI. A whole-brain white matter (skeletonized) mean fractional anisotropy (FA) image is shown. Group differences in diffusion metrics such as fractional anisotropy were assessed using regions of interest, including the subgenual/retrosplenial (red) and parahippocampal (green) subdivisions of the cingulum bundle. (**C**) Functional connectivity was assessed using a dual-regression approach. Two main functional connectivity analyses were performed. One assessed functional connectivity changes between the PCC (yellow) and DMN (blue), the second assessed functional connectivity changes between the PCC and MTL structures, including the hippocampus (red) and parahippocampus (green). ROIS = regions of interest.

Patients were divided into two groups according to performance on the Paired Associate Learning (PAL) task from the Cambridge Neuropsychological Test Automated Battery (CANTAB) computerized tool (Fig. 1A). The PTA group were defined as having PAL scores >2 standard deviations (SD) from the normal mean. Patients with PTA scores <2 SD from the normal mean were defined as TBI controls. As a clinical measure, scores on the Westmead Post-Traumatic Amnesia Scale (WPTAS) were also obtained (Shores *et al.*, 1986) (Supplementary Table 1). The WPTAS is a 12-item scale, with seven items that assess orientation and five items that assess memory. The PAL was preferred as the key PTA classification tool due to the concerns over the validity of the WPTAS in a research context (Marshman *et al.*, 2013). The PAL is: (i) sensitive to memory impairments associated with hippocampal damage (Swainson *et al.*, 2001); and (ii) provides a more detailed and graded assessment of associative memory and learning in comparison to the WPTAS. The PAL thus offers a standardized and validated research tool to assess memory in the context of a hypothesized dysfunction of the MTL subsystem. Correspondence between WPTAS and PAL measures was assessed with a Spearman’s correlation (referred to in the results as ‘rho’) (Supplementary material).

Patients completed neuropsychological testing and scanning once at baseline, all within 15 days of the TBI. Not all patients were able to tolerate the MRI scan on this occasion due to pain or discomfort associated with their head or body injuries. All neuropsychological tasks were completed by the control participants; however, not all tasks were completed by every patient at the acute stage due to fatigue. The intention was for all subjects to return for follow-up assessment. Where possible this was completed once within the first year following injury at a point when memory function had subjectively improved. There was variability in time between baseline and follow-up scans mainly because of variation in clinical recovery, including the resolution of cognitive impairment. To determine whether this variability affected the results, Spearman’s correlations were performed between the time between scans (in months) and the neuropsychological and imaging measures (Supplementary material). A breakdown of the numbers in each analysis (detailed below) is shown in Supplementary Table 2.

#### Control group

Seventeen healthy controls (seven females, mean age 31.3, range 19–49 years) were recruited. All healthy controls completed the neuropsychological testing. Two control participants did not complete the scanning due to MRI contraindications. Participants had no history of psychiatric or neurological illness, previous TBI or alcohol or substance misuse. All participants gave written informed consent. Controls were tested at only one time-point.

### Neuropsychological assessment

**Figure 2:**
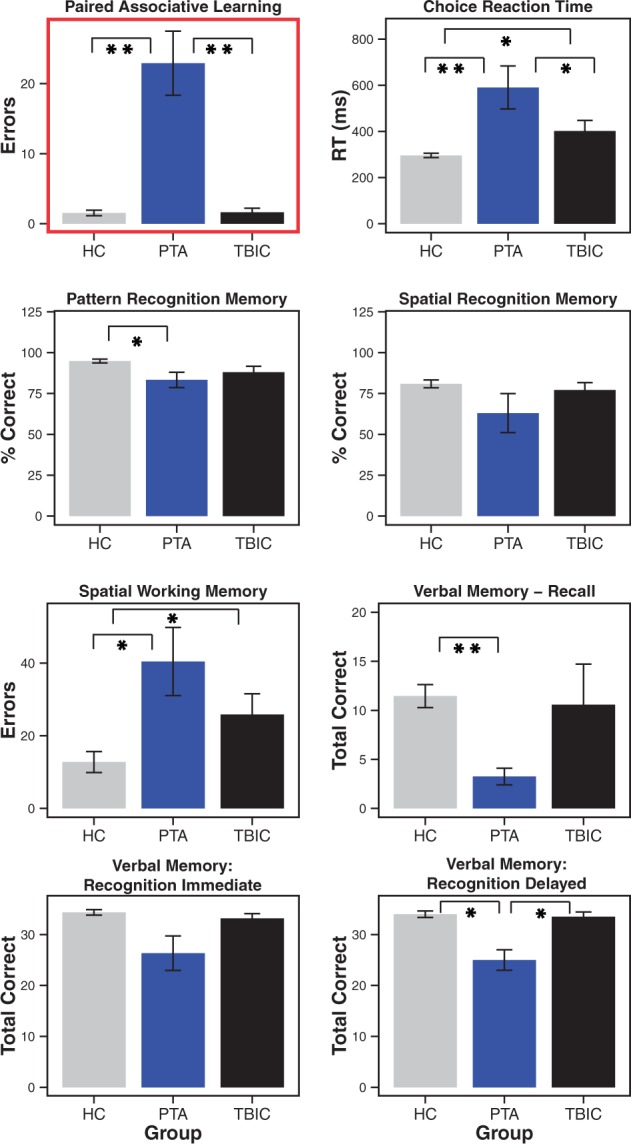
**Neuropsychological results for PTA patients compared to TBI and healthy control groups at baseline.** All tests are derived from the Cambridge Neuropsychological Test Automated Battery (CANTAB) computerized tool. **Significance at *P* < 0.01; *significance at *P* < 0.05. Error bars represent the standard error of the mean (SEM). HC = healthy controls; TBIC = TBI controls; RT = reaction time.

**Figure 3:**
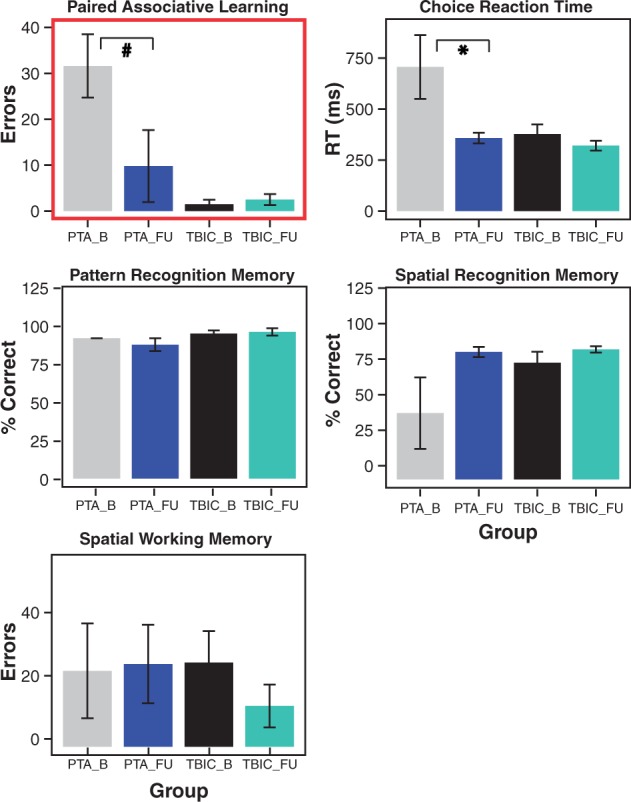
**Neuropsychological results for PTA patients compared to TBI and healthy control groups at follow-up.** All tests are derived from the Cambridge Neuropsychological Test Automated Battery (CANTAB) computerized tool. *Significance at *P* < 0.05, hash symbol indicates a trend. Error bars represent the standard error of the mean (SEM). HC = healthy controls; TBIC = TBI controls; B = baseline; FU = follow-up; RT = reaction time.

The PAL task was used to provide a sensitive measure of associative learning and memory (Fig. 1; see Supplementary material for a detailed description of the PAL task). A standardized neuropsychological battery was used to assess cognitive function more generally. Six tasks from the CANTAB computerized tool were completed. In addition to the PAL, tasks completed consisted of the Choice Reaction Time (CRT) task to assess information-processing speed and sustained attention, the Spatial Working Memory (SWM) task, the Spatial Recognition Memory (SRM) task, the Pattern Recognition Memory task (PRM) task and the Verbal Recognition Memory (VRM) task. A description of the specific outcome measures used for the different tasks can be found in Figs 2 and 3 and Supplementary Table 3. One-way ANOVAs were run to identify group effects at baseline. *Post hoc* independent sample *t*-tests (Welch’s two-sample *t*-test) were performed to determine which pairwise comparisons were driving any significant main effects identified. Linear mixed-effects models were used to assess longitudinal changes between baseline and follow-up. Group and time point were defined as fixed effects, whereas subject was defined as a random effect to model variability in subject intercepts. *Post hoc* paired sample *t*-tests were used to investigate any significant main effects or interactions. Follow-up analyses were not performed on the VRM and PRM tasks for PTA patients due to insufficient data points. All statistical analysis was performed using R (v0.98.1091).

### Structural and functional MRI acquisition

MRI data were obtained using a 3.0 T GE Medical Systems scanner with an 8-channel head coil. Standard clinical MRI was collected. Functional resting state data were collected along with structural MRI data, including a T_1_-weighted high-resolution scan and DTI (see Supplementary material for details on the acquisition parameters).

### Statistical analysis of imaging

Lesion locations were reported by a senior neuroradiologist (Supplementary Fig. 1). Lesion masks were also created to determine lesion size and create overlap images (Supplementary Fig. 2 and Supplementary material).

### Functional MRI: resting state functional connectivity

Data were analysed using the FMRIB Software Library (FSL Version 5.0, Oxford, UK; (Smith *et al.*, 2004)) (see Supplementary material for details on preprocessing).

A dual-regression approach was used to assess functional connectivity differences between control and patient groups (Leech *et al.*, 2011) (Fig. 1C). This approach provides a voxel-wise measure of functional connectivity that represents the temporal correlation between each voxel and the activity of a region or network of interest (Sharp *et al.*, 2011). This method includes three steps: (i) definition of a seed region of interest or network of interest; (ii) use of this region or network to extract individual subject time series; and (iii) re-regression of the extracted time series onto the individual subject’s data to generate a subject-specific spatial map of functional connectivity (Sharp *et al.*, 2011; Ham *et al.*, 2014). The resulting spatial maps were used to compare functional connectivity between patient and control groups. Between-group differences were assessed using non-parametric permutation testing and correction for multiple comparisons was applied using the threshold-free cluster enhancement (TFCE) method and a family-wise error (FWE) rate of *P* < 0.05 (Smith *et al.*, 2004). Due to the focused nature of our hypotheses the group analyses were constrained to voxels within specific regions and networks of interest.

To assess alterations in connectivity within the DMN, including the MTL subsystem, three dual-regression analyses were performed. For these analyses the ventral PCC was chosen as the seed region of interest, defined using an 8 mm diameter spherical mask centred on MNI coordinates (2, −58, 28) taken from a previous connectivity study demonstrating the importance of this specific area in the functionality of the DMN (Leech *et al.*, 2011). The ventral PCC has been shown to have direct anatomical connections to the MTL (Leech *et al.*, 2011). Hence we felt that this location within the DMN was likely to be sensitive to changes in connectivity with the MTL.

The first dual-regression assessed functional connectivity between the ventral PCC and the whole DMN; group-level voxel-wise connectivity analysis was constrained to within a predefined DMN mask, generated using an independent component analysis of 36 healthy control subjects’ resting state data (Smith *et al.*, 2009). This was used to ensure that no group bias was present and included the precuneus, posterior cingulate, temporal lobes and medial prefrontal areas. To specifically assess connectivity between posterior and anterior nodes of the DMN, connectivity between the PCC and vmPFC, was investigated with the use of a targeted region of interest analysis. The vmPFC region of interest was defined using an 8 mm spherical mask centred on the peak coordinate (2, 54, −4) within the Smith *et al.* (2009) DMN. For this region of interest and each subject/visit, region of interest mean functional connectivity values were extracted. A one-way ANOVA was run on the extracted data to identify group effects at baseline. *Post hoc* independent sample *t*-tests (Welch’s two-sample *t*-test) were performed to determine which pairwise comparisons were driving any significant main effects identified.

The second dual-regression was performed to characterize functional connectivity alterations specifically between the ventral PCC and the MTL subsystem of the DMN. PCC connectivity to two regions within the MTL, the hippocampus and parahippocampus, was assessed by constraining group-level voxel-wise statistical analyses to these two regions. These anatomically-defined regions of interest were derived using the FSL Harvard-Oxford atlas using probabilistic regions thresholded at 20%. These more focused analyses were supplemented by a third set of dual-regressions. These included analyses performed with the use of: (i) a visual network thought to be unaffected by PTA; and (ii) brain networks involved in higher order cognition—bilateral fronto-parietal networks and the executive control network defined from Smith *et al.* (2009) (Supplementary material and Supplementary Fig. 3).

Dual-regression analyses were performed at baseline and follow-up. In addition, areas identified as showing group-level altered functional connectivity at baseline, including the precuneus, parahippocampus and vmPFC, were used to determine whether connectivity normalized at follow-up. Linear mixed-effects models were used to assess these longitudinal effects. Group (PTA and TBI controls) and time point were defined as fixed effects, whereas subject was defined as a random effect to model variability in subject intercepts. *Post hoc* paired sample *t*-tests were used to investigate any significant main effects or interactions. The significant functional connectivity changes at baseline were correlated with neuropsychological data to investigate whether individual differences in connectivity were associated with the extent of cognitive impairment using Spearman’s correlation as the data were non-parametrically distributed.

### Structural MRI connectivity: diffusion tensor imaging

Standard FSL approaches to DTI analysis were used to obtain voxel-wise individual subject fractional anisotropy, mean diffusivity, axial diffusivity and radial diffusivity maps (Supplementary material). A region of interest approach was used to assess between-group differences in white matter integrity. Two regions of interest were defined within the cingulum bundle in both the right and left hemisphere (four in total). The first region of interest combines the subgenual and retrosplenial subdivisions of the cingulum bundle that link two primary nodes of the DMN, the PCC and vmPFC. The second region of interest includes the restricted parahippocampal subdivision of the cingulum bundle that connects the PCC to the parahippocampal cortices (Jones *et al.*, 2013) (Fig. 1B). This structural analysis allowed us to focus on white matter connections within the core DMN (subgenual/retrosplenial subdivision) and between the core DMN and MTL subsystem (parahippocampal subdivision), complementing the functional connectivity analysis. Based on previous results demonstrating the relationship between structural integrity of the fornix and associative learning and memory (Kinnunen *et al.*, 2011), this tract was also used as an additional region of interest. All regions of interest were within the John Hopkins University White-Matter Tractography and Juelich Histological atlases available within FSL. For each region of interest and each subject/visit, region of interest mean summary values (fractional anisotropy, mean diffusivity, axial diffusivity and radial diffusivity) were extracted. One-way ANOVAs were run on the extracted data to identify group effects at baseline. *Post hoc* independent sample *t*-tests (Welch’s two-sample *t*-test) were performed to determine which pairwise comparisons were driving any significant main effects identified. Changes in white matter integrity between baseline and follow-up were assessed with the use of paired-sample *t*-tests. Whole-brain analyses were also performed for exploratory purposes. Statistical assessments were performed using non-parametric permutation testing with the TFCE method and a threshold of *P* < 0.05 to correct for multiple comparisons. These analyses were completed both at baseline and follow-up time points.

## Results

### Neuropsychological performance

#### Patient classification

Patients were divided into two groups based on their performance on the PAL task (Fig. 2). Eleven patients were classified as having PTA [two females, mean age 40 years (range 28–61), mean time since injury 5.73 days (2–14 days)]. A TBI control group of eight TBI patients performed in the normal range on the PAL [one female, mean age 37.1 years (range 27–60), mean time since injury 5.38 days (1–13 days)]. The control and patient groups did not differ significantly in age [*F*(2,33) = 2.19, *P* = 0.129], time since injury or injury severity.

#### Neuropsychological performance at baseline

As expected from the way the two patient groups were defined, the PTA group showed evidence of highly significant memory impairment as measured by the PAL task. A significant effect of group resulted from increased error rates in the PTA group relative to the TBI control [*t*(10.34) = 4.61, *P* < 0.001] and healthy control groups [*t*(10.14) = −4.65, *P* < 0.001]. There was no difference between the TBI control and healthy control groups [*t*(13.06) = −0.13, *P* = 0.447] (see Supplementary Table 3 for statistics).

The PTA group also showed significant impairments in other aspects of cognitive function (Fig. 2, Supplementary Table 3 and Supplementary material). Relative to both control groups, PTA patients were significantly impaired on the choice reaction time, spatial working memory, and delayed verbal recognition memory tasks. Pattern recognition memory accuracy was impaired only compared to healthy controls, whereas reaction time measures associated with this task were significantly slower compared to both control groups. No significant impairments in spatial recognition memory accuracy were present, while reaction times associated with this task were marginally affected. Immediate verbal recognition demonstrated a trend towards impairment. Free verbal recall was unaffected (see Fig. 2, Supplementary Table 3 and Supplementary material for statistics).

#### Longitudinal changes in neuropsychological performance

As expected, there was a general improvement in cognitive function in the PTA group over time Fig. 3 and Supplementary Table 3). The number of patients who returned for follow-up assessment was ∼50%, a rate in keeping with this type of clinical study whereby patients are recruited in an acute setting. Associative memory function generally improved at follow-up. A significant effect of group was seen, with effects of time point and group by time point interaction of borderline significance. These changes were the result of improvement in the PTA group [*t*(4) = 1.97, *P* = 0.06] with no change in TBI control group performance [*t*(3) = −0.51, *P* = 0.68]. A similar pattern of significant effects was seen in pattern recognition memory reaction times, although *post hoc* tests were not significant (Supplementary Table 3). Information processing speed, indexed by the CRT task, demonstrated a significant effect of time point (Supplementary Table 3). Reaction times were slower at baseline across both groups. The PTA group improved significantly over time [*t*(4) = 2.19, *P* = 0.047], which was not seen in the TBI control group [*t*(2) = 0.62, *P* = 0.30; Fig. 3]. There were no other significant longitudinal changes.

### TBI is associated with altered connectivity within default mode network

**Figure 4:**
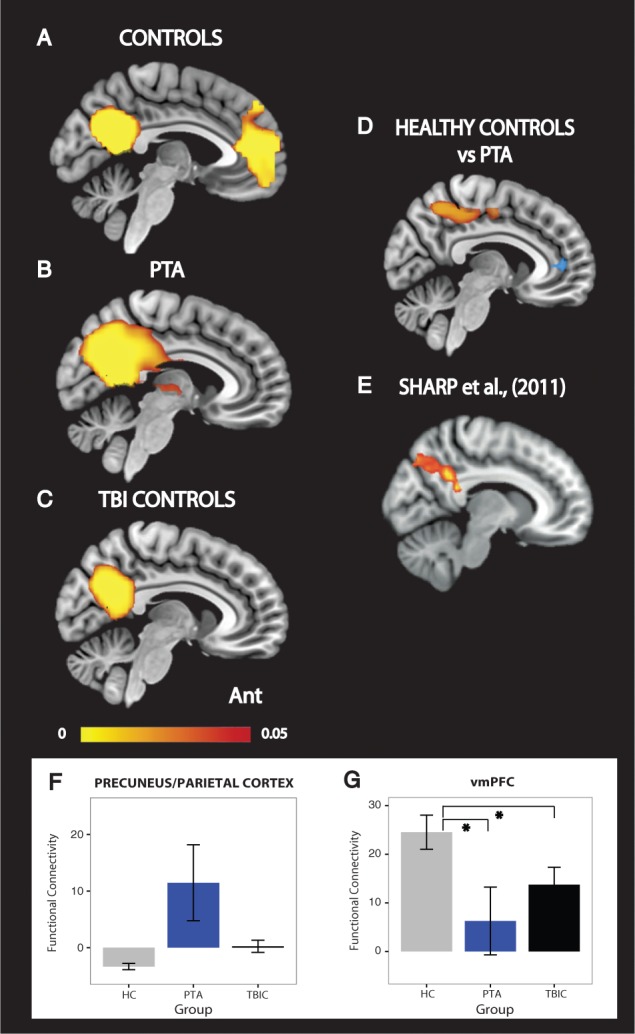
**Functional connectivity within the DMN.** Functional connectivity of the PCC to the rest of the DMN in (**A**) healthy controls (HC), (**B**) PTA patients and (**C**) TBI controls. (**D**) The direct contrast between PTA patients and healthy controls. Yellow-red colours indicate areas of increased functional connectivity in PTA patients compared to healthy controls. Blue areas indicate brain areas of reduced functional connectivity to the PCC in PTA patients compared to healthy controls. Results are overlaid on the MNI152 T_1_ 1 mm brain template. (**E**) Voxels showing greater functional connectivity with a DMN-specific time-course in an independent cohort of patients following TBI (Sharp *et al.*, 2011). (**F** and **G**) Graphs representing PCC functional connectivity changes in precuneus/parietal cortex and vmPFC. The precuneus/parietal graph is presented for visualization purposes alone. Ant. = anterior; TBIC = TBI controls. The colour bar represents *P*-values in the range of 0 to 0.05. All connectivity maps are significant at *P* < 0.05, family-wise error (FWE) except for the area of reduced functional connectivity in blue. This is displayed at an uncorrected threshold. *Significance at *P* < 0.05. Error bars represent the standard error of the mean (SEM).

Functional connectivity within the DMN was abnormal following TBI (Fig. 4), extending previous work demonstrating a similar result (Fig .4E). We examined functional connectivity of the central node in the DMN, the PCC, to the rest of the DMN. Healthy controls demonstrated a typical pattern of PCC functional connectivity, including DMN areas such as the precuneus and vmPFC (Fig. 4A). This specific pattern of connectivity was not apparent in either of the patient groups (Fig. 4B and C). At a voxel-wise level, directly comparing the PTA and healthy control groups showed significantly increased functional connectivity from the PCC to the precuneus and lateral parietal parts of the DMN in PTA patients (Fig. 4D). In contrast, PCC functional connectivity to the vmPFC showed a trend to being abnormally low compared to controls, although this result did not survive correction for multiple comparisons (Fig. 4D and G). A planned region of interest analysis of PCC functional connectivity to the vmPFC confirmed a significant group effect [*F*(2,27) = 4.27, *P* = 0.024], driven by significantly lower functional connectivity in both the PTA [*t*(10.64) = 2.34, *P* = 0.039] and TBI control [*t*(16.44) = 2.15, *P* = 0.046] groups compared to healthy controls, representing a non-specific change across the TBI patients. No significant differences in functional connectivity were found between the PTA and TBI control groups. Mixed-effects models including baseline and follow-up time points showed no statistically significant change in DMN connectivity over time (Figs 4D and 6).

### PTA is associated with reduced connectivity between the default mode network and parahippocampus, which normalizes with recovery

**Figure 5:**
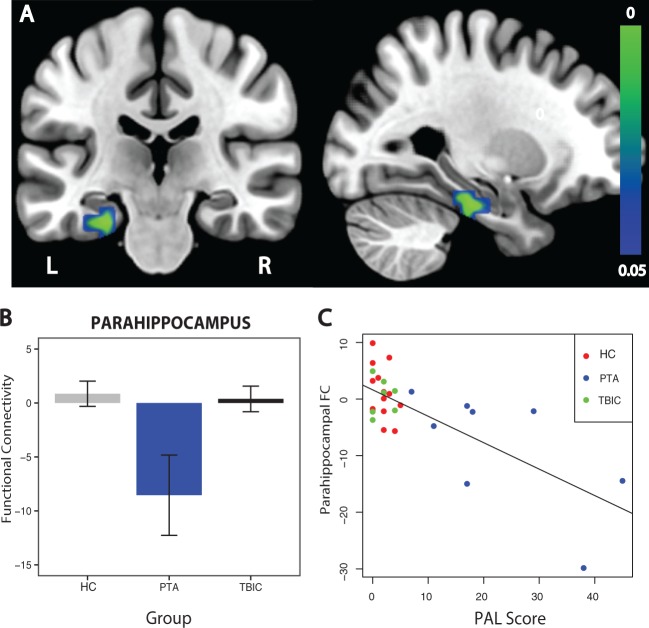
**Functional connectivity between the PCC and parahippocampus.** (**A**) Direct contrast between PTA patients and healthy controls. Results are overlaid on the MNI152 T_1_ 1 mm brain template. (**B**) Graph representing functional connectivity (FC) values for the three experimental groups extracted from the parahippocampal brain area demonstrating significantly reduced functional connectivity in the direct contrast between PTA patients and healthy controls (HC). (**C**) Graph representing a significant correlation between PCC functional connectivity to the parahippocampus and scores on the PAL task. The colour bar represents *P*-values in the range of 0 to 0.05. L = left; R = right; TBIC = TBI controls.

We next specifically investigated the functional connectivity between the PCC and MTL structures (Fig. 5). At a voxel-wise level, functional connectivity between the PCC and parahippocampus was significantly reduced in the PTA group compared to the healthy control group (Fig. 5A and B). The region of reduced connectivity was located on the border of the anterior and posterior subdivisions of the left parahippocampus (Fig. 5A). TBI controls showed no difference in functional connectivity compared to healthy controls and there were no group differences in hippocampal functional connectivity at the voxel-wise level.

**Figure 6:**
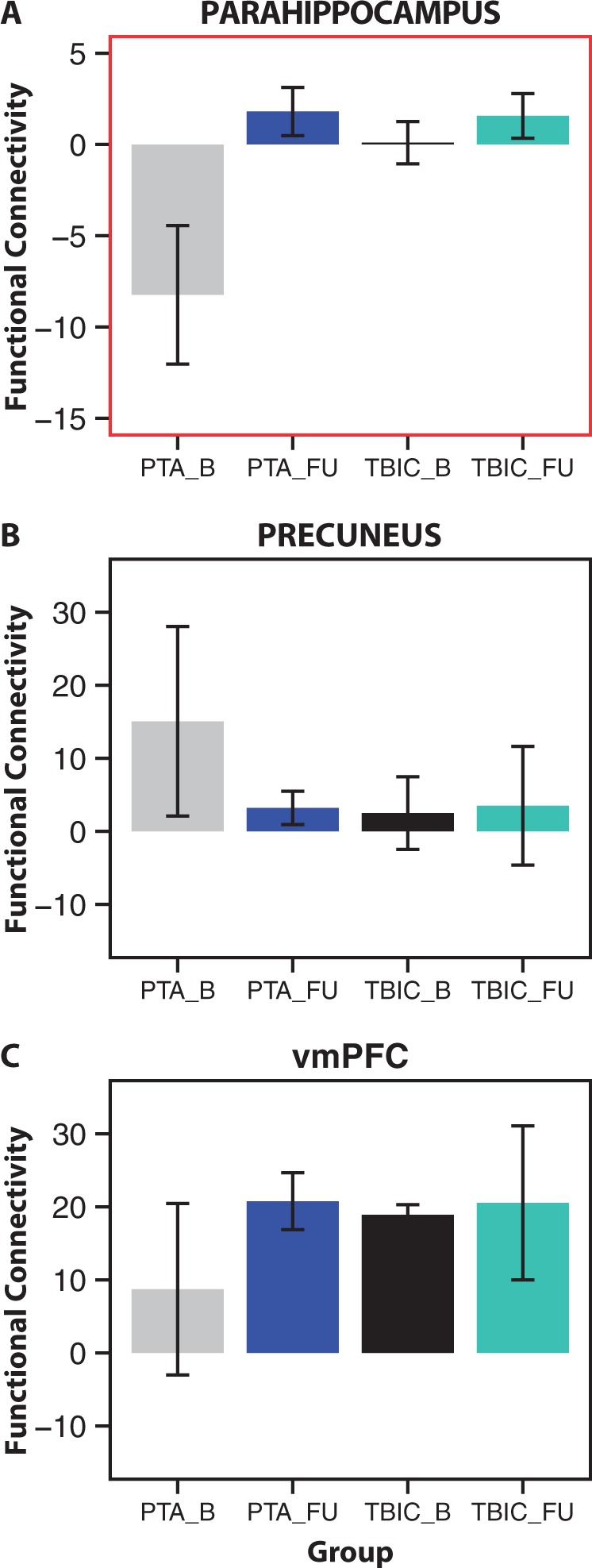
**Functional connectivity between the parahippocampus and PCC normalizes at follow-up.** Functional connectivity of the PCC to the parahippocampus (**A**), precuneus (**B**), and vmPFC (**C**) at baseline and follow-up. HC = healthy controls; TBIC = TBI controls; B = baseline; FU = follow-up.

At follow-up, the PTA group showed a trend towards normalization of parahippocampus functional connectivity (Figs 5A and 6). A mixed-effects model showed an overall increase in functional connectivity with time [*F*(1,11) = 5.699, *P* = 0.036]. This was largely the result of a normalization of functional connectivity in the PTA group, which was of borderline significance [*t*(3) = −1.97, *P* = 0.072]. In contrast, functional connectivity remained stable in TBI controls [*t*(2) = −0.76, *P* = 0.53]. The time point by group interaction was not significant [*F*(1,10) = 2.988, *P* = 0.115] and there was no main effect of group [*F*(1,11) = 2.254, *P* = 0.161].

### Parahippocampal functional connectivity correlates with memory function

Decreased connectivity between the PCC and parahippocampus was associated with increasing impairments in associative memory. Individual differences in parahippocampal connectivity were significantly correlated with performance on the PAL task across all subjects (rho = −0.46, *P* = 0.0098; Fig. 5C), as well as when patients were assessed alone (rho = −0.57, *P* = 0.028; Fig. 5C). To assess whether this relationship was specific to PAL performance, correlations were performed with the additional neuropsychological tasks. Decreases in parahippocampal connectivity were also significantly correlated with longer reaction times on pattern recognition memory performance both across all subjects (rho = −0.417, *P* = 0.03) and in patients alone (rho = −0.72, *P* = 0.008). This measure also correlated with spatial working memory performance (rho = −0.52, *P* = 0.005) and immediate verbal recognition memory (rho = 0.48, *P* = 0.045), although not significantly when the patient group was studied alone (rho = −0.48, *P* = 0.112; rho = 0.55, *P* = 0.157).

### Additional brain network analyses

No functional connectivity changes within the PTA group were observed within the visual network. In contrast, the fronto-parietal and executive control networks did show significant changes in connectivity in the PTA group at baseline (Supplementary material and Supplementary Fig. 3). The right fronto-parietal network showed increases in connectivity to a wide range of regions with peak changes in the middle frontal and post-central gyri, whereas the left fronto-parietal network showed peak increases in connectivity with the precuneus (Supplementary Fig. 3A and B). The PTA group also showed increases in connectivity within the executive control network, with peak increases in cingulo-opercular and inferior frontal regions (Supplementary Fig. 3C). Decreases in connectivity from the executive control network to the hippocampus and cerebellum were also seen within the PTA group (Supplementary Fig. 3C). Only changes in the executive control network showed normalization of functional connectivity at follow-up (Supplementary Fig. 3D). PCC connectivity to these networks was altered, although this was seen mainly across both patient groups (see Supplementary material for detailed results).

### Motion

Motion across all participants was minimal (<0.5 mm in relative root mean squared frame-wise displacement; RMSFD). PTA patients and TBI controls demonstrated an average relative RMSFD of 0.16 mm and 0.12 mm, respectively, compared to 0.06 mm in healthy controls (see Supplementary material for further discussion of this issue).

### PTA is associated with reduced white matter integrity in the parahippocampal subdivision of the cingulum bundle

**Figure 7:**
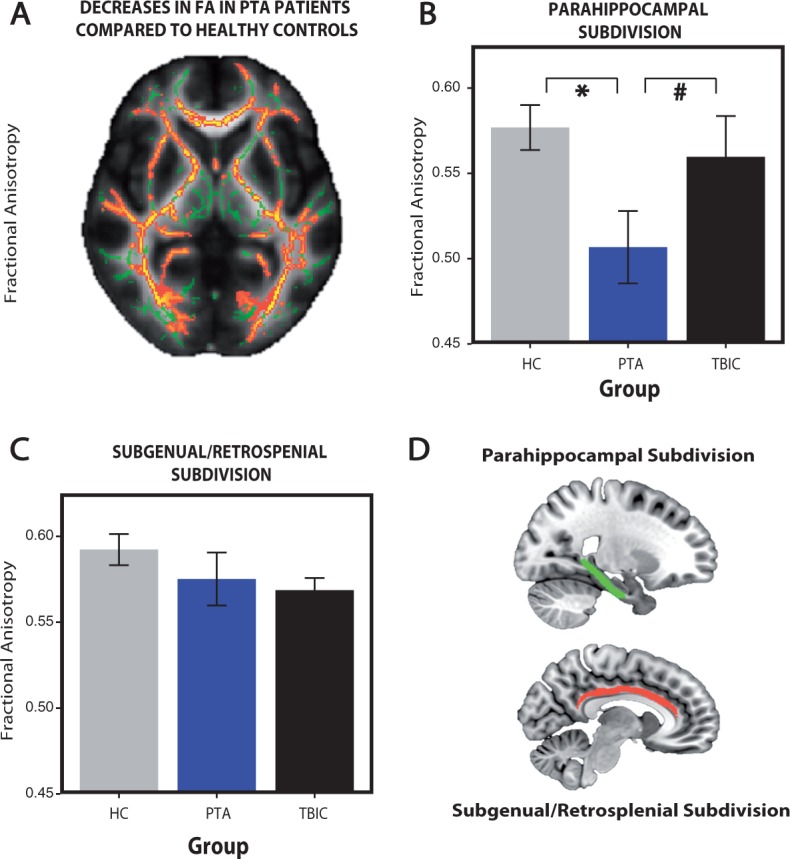
**Reduced white matter integrity in PTA patients compared to healthy controls.** (**A**) Fractional anisotropy (FA) reductions in whole-brain white matter (skeletonized) from a direct contrast between the healthy controls and PTA patients. Green = normal white matter; red = damaged areas (low fractional anisotropy). Fractional anisotropy changes within the parahippocampal (**B**) and subgenual/retrosplenial (**C**) subdivisions of the cingulum bundle. (**D**) Parahippocampal (red) and subgenual/retrosplenial (green) subdivisions of the cingulum bundle. *Significance at *P* < 0.05, hash symbol indicates a trend. Error bars represent the standard error of the mean (SEM). HC = healthy controls; TBIC = TBI controls.

As expected, DTI provided evidence of white matter disruption in PTA patients. A whole-brain analysis showed widespread reductions fractional anisotropy, characteristic of diffuse axonal injury (Fig. 7A); in comparison to healthy controls, the PTA group showed areas of significant reductions in fractional anisotropy including the genu and splenium of the corpus callosum, anterior and posterior limbs of the internal capsule, external capsule, anterior and superior corona radiata, posterior thalamic radiation and uncinate fasciculus (Fig. 7A). At the whole brain level, there were no significant differences between healthy and TBI controls nor between PTA patients and TBI controls. No significant whole-brain differences were found for mean diffusivity, radial diffusivity or axial diffusivity.

We specifically examined diffusion metrics within two subdivisions of the cingulum bundle. At baseline, the PTA group showed reduced fractional anisotropy within the right parahippocampal subdivision of the cingulum bundle. A significant group effect [*F*(2,25) = 3.70, *P* = 0.039] was present, driven by reduced fractional anisotropy in the PTA group in comparison to both healthy [*t*(9.16) = 2.81, *P* = 0.01] and a borderline difference to the TBI control groups [*t*(9.86) = −1.66, *P* = 0.065] (Fig. 7B). A similar pattern of results was seen with radial diffusivity. A significant group effect [*F*(2,25) = 4.77, *P* = 0.018] was driven by significantly increased radial diffusivity in the PTA group in comparison to healthy controls [*t*(8.38) = −2.94, *P* = 0.009] and a trend difference with TBI controls [*t*(9.98) = 1.40, *P* = 0.096]. No significant changes were seen in axial diffusivity. Both PTA patients and TBI controls showed increases in mean diffusivity. ANOVA showed a significant effect of group [*F*(2,25) = 5.23, *P* = 0.013], driven by significant increases in mean diffusivity in PTA patients [*t*(9.24) = −2.63, *P* = 0.013] and TBI controls [*t*(16.92) = −3.08, *P* = 0.003] compared to healthy controls. In contrast, the left parahippocampal and bilateral subgenual/retrosplenial subdivisions of the cingulum bundle, did not show any significant evidence of damage at a group level (Fig. 7C).

In addition, we examined the main outflow tract of the hippocampus, the fornix, which showed no significant evidence of damage across all diffusion metrics at a group level. At baseline, the extent of white matter damage in patients within the right parahippocampal subdivision was correlated with memory performance measured by the PAL, as indexed by fractional anisotropy (rho = −0.68, *P* = 0.015) (Fig. 7D). Longitudinal analysis of the diffusion data did not yield any significant results, at the whole-brain or region of interest-based level, although this could be the result of the small sample size available for these analyses (five patients; Supplementary Table 2).

## Discussion

PTA is common after TBI. Despite this, the biological basis of this phenomenon is uncertain, limiting our understanding of the early effects of TBI. Our results show for the first time that PTA is associated with disruption to the structure and function of brain networks critical for memory formation. The PCC is the main hub of a network central to memory processing, the DMN. This network comprises a number of subsystems, including the medial temporal subsystem, which incorporates hippocampal and parahippocampal structures (Andrews-Hanna *et al.*, 2014). We show: (i) impairments in associative memory in patients with PTA were accompanied by deficits in information processing speed and spatial working memory; (ii) both structural and functional connectivity from the PCC to the parahippocampus is disrupted when patients are in PTA; (iii) the extent of the disruption in functional and structural connectivity is correlated with episodic memory impairment and a measure of information processing speed; (iv) the resolution of these impairments clinically is associated with a normalization of this physiological change; and (v) connectivity changes in fronto-parietal and executive control networks are also present during PTA, reflecting a widespread disruption in intrinsic connectivity network involved in supporting cognitive function. The work provides preliminary evidence that disruption to the functional interactions between nodes of the DMN is central to the profound disturbance in memory function seen in PTA.

The alterations in functional connectivity we have observed are likely to reflect disruption to oscillatory synchrony across brain networks (Nyhus and Curran, 2010). This is relevant to memory function as fluctuations in network synchrony are proposed to support complex cognitive processes including memory encoding, consolidation and retrieval (Duzel *et al.*, 2010; Nyhus and Curran, 2010). Work in non-human primates strikingly demonstrates that the consolidation of new memories is associated with coordinated activity between MTL structures and the neocortex, with the largest changes in cortical activity linked to fast hippocampal oscillations (ripples) seen in the PCC and adjacent retrosplenial cortex (Logothetis *et al.*, 2012). Disruption to these interactions, and accompanying memory consolidation, occurs if these ripples are suppressed (Girardeau and Zugaro, 2011). In humans, theta oscillations between the posterior parietal cortex and the parahippocampus synchronize during associative encoding, suggesting that functional disconnection to the parahippocampus could lead to impairments with associative memory, a pattern of results seen in this study (Crespo-Garcia *et al.*, 2010).

We showed a transient impairment in interactions between the parahippocampus and the PCC during PTA. This may reflect a role for the parahippocampus in mediating interactions between the hippocampus and neocortical regions, particularly the PCC. The parahippocampus shows strong functional connectivity with the PCC in the absence of explicit task demands (Ward *et al.*, 2014) and abnormalities in functional connectivity between the MTL structures and the PCC have been shown to be associated with memory impairment in amnesic patients following damage to bilateral MTL (Hayes *et al.*, 2012). A strength of our study is that we were able to investigate a subset of patients after they exited from PTA. This allowed us to test whether the network abnormalities observed in the acute setting normalized with recovery. The parahippocampal functional connectivity to the PCC normalized once patients were able to encode new memories, providing evidence that the abnormality we observed may be the physiological basis for the transient amnestic impairment seen in PTA.

Abnormal functional connectivity between the PCC and parahippocampus was associated with both memory and information processing impairments, suggesting that these changes are important for cognitive functions other than memory. These results extend our previous work showing that functional abnormalities within the DMN are commonly seen in the chronic phase and relate to attentional impairments, which may explain the processing speed changes seen (Sharp *et al.*, 2011). We also observed functional connectivity abnormality within higher-order fronto-parietal and executive control networks demonstrating a widespread disruption of brain network function. This was correlated with a measure of processing speed associated with pattern recognition memory. This suggests that changes to the interactions of the DMN with the MTL subsystem may be relevant to particular aspects of memory function, such as the formation of associations, whereas disruption to other networks may influence distinct processes that influence other aspects of cognition.

It is perhaps surprising that we found no evidence for a significant alteration in hippocampal functional connectivity in PTA patients, considering the role of hippocampal connectivity in memory formation (Ranganath *et al.*, 2005). However, this could be explained by the way MTL structures are connected to the PCC. The parahippocampal subdivision of the cingulum bundle connects the medial temporal lobe to the PCC and terminates predominantly within the parahippocampus rather than the hippocampi (Squire *et al.*, 2004; Parvizi *et al.*, 2006; Schmahmann *et al.*, 2007). This structural distinction is reflected in distinct patterns of functional connectivity, whereby interactions between the hippocampus and PCC are mediated through the parahippocampus (Ward *et al.*, 2014). Hence, our differential results between the PCC and the hippocampi may simply reflect the underlying connections of these structures.

We found evidence of widespread white matter damage in the PTA group, indexed by both fractional anisotropy and radial diffusivity changes. Specific damage to the parahippocampal subdivision of the cingulum bundle that connects the MTL to the PCC was also found. Hence, the alteration in functional connectivity we observed might relate to diffuse structural changes or a more specific injury to connections within limbic structures. The importance of the parahippocampal subdivision to memory function is supported by the observation that abnormalities within this tract are also seen in disorders such as Alzheimer’s disease (Zhang *et al.*, 2007; Fakhran, *et al.*, 2013), where both functional disconnection between the DMN and MTL and memory impairments are evident (Wang *et al.*, 2006; Dunn *et al.*, 2014). However, group differences in fractional anisotropy were observed in the right cingulum bundle, whereas the functional connectivity abnormality was observed in the left parahippocampus. Hence, the relationship between structural damage and changes in functional connectivity is likely to be complex and will require more investigation in a larger cohort of patients.

We also showed that functional connectivity to other parts of the DMN was disrupted following TBI. Within posterior parts of the DMN (PCC, precuneus and lateral parietal regions), we observed an increase in functional connectivity. We have previously observed a similar increase in PCC connectivity in TBI patients with persistent problems following TBI (Sharp *et al.*, 2011). In contrast, we observed a decrease in functional connectivity to the ventromedial PFC, again a result that has been seen in other contexts, such as ageing-associated cognitive decline (Andrews-Hanna *et al.*, 2007). Furthermore, coupling strength between posterior and anterior regions of the DMN has previously been shown to facilitate memory performance (Hampson *et al.*, 2006). These findings suggest that distinct breakdown in functional connectivity within subregions of the DMN may be associated with memory impairment, with increased parietal connectivity and decreased fronto-parietal connectivity associated with reduced memory performance.

An important consideration in functional connectivity studies is that of subject movement during data acquisition. Very small amounts of motion were seen in our patients, so we do not think that this is a significant factor in explaining our results. We did observe a small difference in movement between healthy controls and patients. However, we took a number of steps to ensure that physiological motion was taken into account during data analysis, including regressing out the effects of motion and performing a subsidiary analysis where one patient with larger degrees of movement was removed. One other potential limitation is the effect of medication on the results. PTA patients are often under the influence of opioids or anticonvulsants, medications that may impair cognitive function (Marshman *et al.*, 2013). In addition, different medications can affect the MRI signal, through alterations in vascular reactivity or neuronal activity (Wise and Tracey, 2006). It is unlikely, however, that the medications are driving the changes in functional connectivity. The TBI control and PTA patient groups did not differ in terms of medications taken, suggesting that differences in neuropsychological deficits and connectivity found are not driven by pharmacological or purely vascular effects. One potential confound is age-related changes in brain structure and function. For example, brain maturation changes might be present in younger participants, whereas older participants may have latent microvascular or cognitive changes that affect brain connectivity (Barnea-Goraly *et al.*, 2005; van Duinkerken *et al.*, 2009). However, age ranges across all experimental groups were well matched and our participants were not older than 61, an age prior to which significant cognitive decline or vascular disease is unlikely (Schaie and Willis, 2010). Furthermore, there was no evidence of vascular disease on clinical imaging. Such factors mitigate the potential impact of age-related changes on the results of this study. A further limitation of this study is its sample size, which might potentially limit the extent to which our findings can be extrapolated across TBI patients. Whilst there is significant heterogeneity in the neuropathologies seen after head injury, it is striking how consistently PTA is seen across patients following a significant head injury. This suggests the presence of a consistent cognitive syndrome, which is likely to have a common underlying physiological basis. Therefore, our results are likely to have broad applicability across TBI patients.

In summary, our results provide novel insights into the pathophysiology of PTA, although it will be important to replicate these findings in a larger cohort of patients. Future work will be needed to clarify the specificity of some of the findings to PTA and to ascertain whether patients with memory impairment following TBI should more accurately be viewed on a continuum, rather than attempting to separate patients into those with and without PTA. Along with clinical assessment of PTA, we provide a full neuropsychological profile, demonstrating that impairments in associative memory were accompanied primarily by information processing and spatial working memory deficits. We demonstrate a transient functional disconnection between the parahippocampus and limbic structures within the DMN during PTA. This disconnection correlated with the degree of memory and attentional dysfunction and normalized when patients exited PTA. Disruption to fronto-parietal and executive control cognitive networks was also found, reflecting the more widespread nature of the cognitive deficits in PTA. Our PTA patients showed evidence of widespread abnormalities in white matter structure, including within the cingulum bundle that connects nodes within the DMN. Hence, axonal dysfunction following TBI may underlie the functional connectivity abnormalities we have observed.

## Supplementary Material

Supplementary Data

## Abbreviations

DMN: default mode network
MTL: medial temporal lobe
PAL: paired associates learning
PCC: posterior cingulate cortex
PTA: post-traumatic amnesia
TBI: traumatic brain injury
vmPFC: ventromedial prefrontal cortex
