# Supplementary material for: Disconnection between the default mode network and medial temporal lobes in post-traumatic amnesia
Source: Brain. 2016 Oct 22;139(12):3137–50. doi: 10.1093/brain/aww241 (PMC5382939; doi:10.1093/brain/aww241)
Supplement: Supplementary Data [file aww241_supp.zip › brain-2015-02273-File012.pdf]

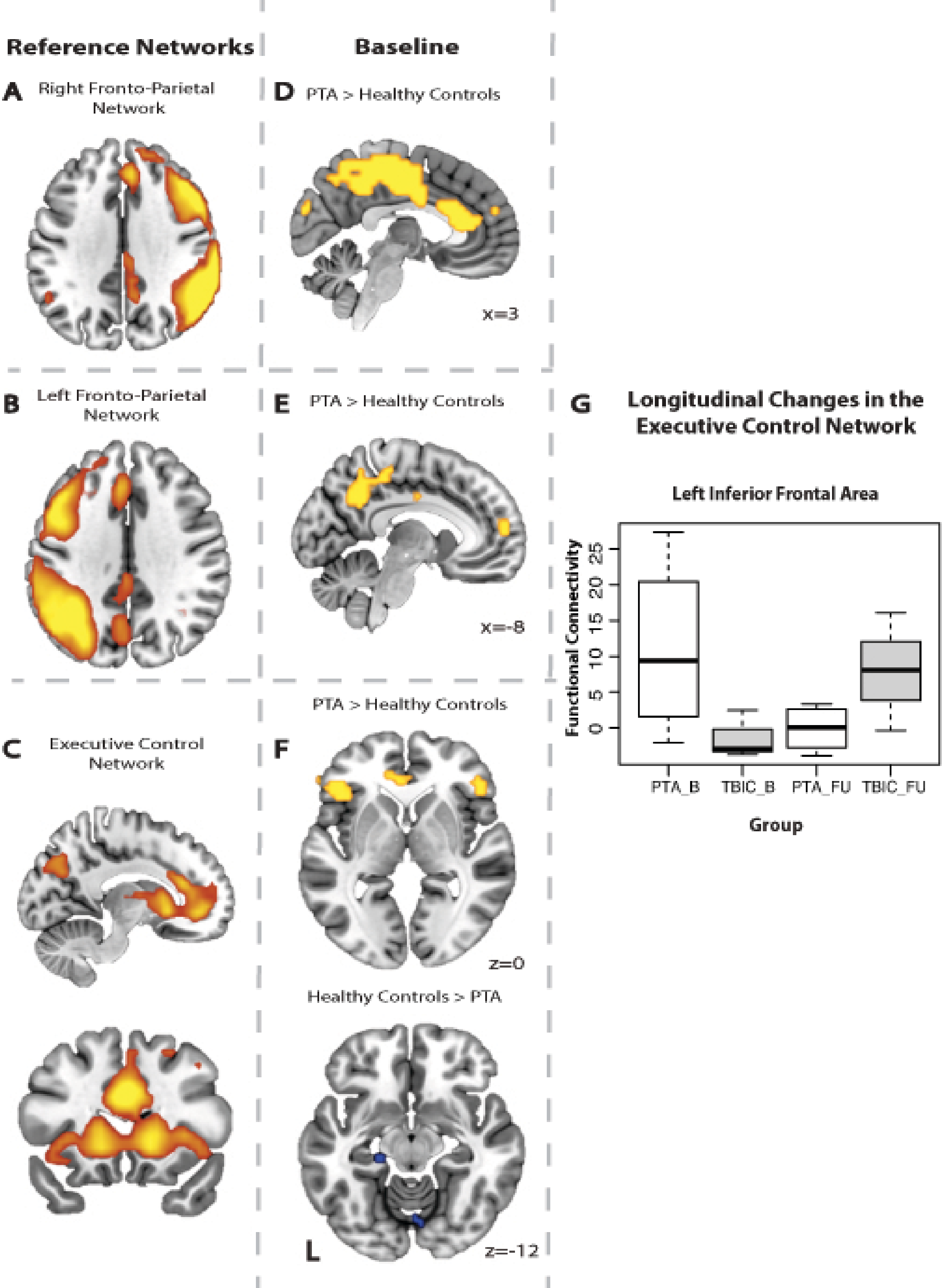

Reference Networks
Baseline
A
Right Fronto-Parietal Network
D
PTA > Healthy Controls
x=3
B
Left Fronto-Parietal Network
E
PTA > Healthy Controls
x=-8
G
Longitudinal Changes in the Executive Control Network
Left Inferior Frontal Area
Functional Connectivity
0
5
10
15
20
25
PTA_B
TBIC_B
PTA_FU
TBIC_FU
Group
C
Executive Control Network
PTA > Healthy Controls
F
z=0
Healthy Controls > PTA
L
z=-12
