# Supplementary material for: Disconnection between the default mode network and medial temporal lobes in post-traumatic amnesia
Source: Brain. 2016 Oct 22;139(12):3137–50. doi: 10.1093/brain/aww241 (PMC5382939; doi:10.1093/brain/aww241)
Supplement: Supplementary Data [file aww241_supp.zip › brain-2015-02273-File013.pdf]

PAL Group	Westmead PTA Score	PTA Duration	Age	Sex	Severity	Cause	LOC Duration	Medication at the time of scanning	Structural MR findings at Baseline
PTA	11	Ongoing	52	M	Mod/Sev	Fall	Unknown	Nil	Bilateral anterior and inferior temporal lobe haemorrhagic contusions.<br>CT Scan – Diffuse injury
PTA	11	Ongoing	52	M	Mod/Sev	Fall	Unknown	Paracetamol 1mg PRN<br>Dihydrocodeine 30mg PRN<br>Cyclizine 50mg PRN<br>Phenytoin 300mg OD	Right frontal, parietal and lateral temporal lobe contusions. Right gyrus rectus contusions. Right cerebral convexity subdural haematoma extending into the middle cranial fossa and overlying the tentorium. Extra-dural and extra-axial haematoma overlying left temporal lobe.<br>CT Scan – Mass lesions
PTA	11	Ongoing	30	M	Mod/Sev	RTA	1-29 minutes	Paracetamol 1g QDS<br>Dihyrocodeine 60mg QDS<br>Oramorph 20mg QDS<br>Lactulose PRN	Bilateral subfrontal and left temporal pole contusions. Right parafalcine frontal lobe, left putamen, globus pallidus, left thalamus, left mammilothalamic tract and red nuclei microbleeds.<br>CT Scan – Diffuse injury
PTA	10	Ongoing	28	F	Mod/Sev	RTA	1-29 minutes	Co-Amoxiclav 1.2g TDS	N/A<br>CT Scan – Mass lesions

								Docusate 200mg OD<br>Lansoprazole 30mg OD<br>Paracetamol 1g PRN<br>Phenytoin 300mg OD<br>Senna 1 tablet<br>Dihydrocodeine 60mg QDS<br>Oramorph 10mg PRN<br>Cyclizine 50mg PRN<br>Ondansetron 4mg TDS	
PTA	9	Ongoing	39	M	Mod/Sev	Fall	Unknown	Dihydrocodeine 60mg QDS<br>Paracetamol 1g QDS	N/A<br>CT Scan – Diffuse injury
PTA	3	Ongoing	33	F	Mod/Sev	Fall	Unknown	Paracetamol 1g QDS	Bilateral medial temporal lobe and splenium haemorrhagic contusions.

									Right medial temporal contusion extends into the temporal uncus. Left ventral thalamus, right splenium and left inferior ganglionic microhaemorrhages.<br>CT Scan – Diffuse injury
PTA	12	1-29 minutes	28	M	Mod/Sev	RTA	Unknown	None.	Normal scan.<br>CT Scan – Diffuse Injury, NVP
PTA	12	1-7days	58	M	Mod/Sev	RTA	1-7days	Paracetamol 1g QDS<br>Lansoprazole 30mg OD<br>Senna 2 tablets BD<br>Docusate 200mg BD	Frontoparietal and left temporal lobe haemorrhagic contusions.<br>CT Scan - Diffuse Injury
PTA	12	Unknown	31	M	Mod/Sev	Fall	Unknown	Pabrinex I+II TDS<br>Paracetamol 1g QDS<br>Dihydrocodeine 60mg TDS<br>Phenytoin 300mg OD	Left lateral temporal lobe haemorrhagic contusions. Left frontal subdural haematoma, extending over the left side of the tentorium.<br>CT Scan – Mass lesions
PTA	11	Unknown	28	M	Mod/Sev	Assault	Unknown	Phenytoin 300mg	N/A

								Paracetamol PRN<br>Dihydrocodeine 60mg QDS<br>Ibuprofen 400mg TDS	CT Scan – Mass lesions
PTA	12	1-7 days	61	M	Mod/Sev	RTA	No	Paracetamol 1g PRN<br>Lansoprazole 30mg<br>Gabapentin 600mg TDS<br>Prochlorpherazine 10mg TDS<br>Slow sodium 1.2g BD	Subacute right frontal haematoma. Subdural haematomas overlying both frontal and occipital lobes.<br>Left inferior frontal gyrus haemorrhagic contusions.<br><br>CT Scan – Mass lesions
TBIC	10	Ongoing	36	M	Mod/Sev	RTA	Unknown	Paracetamol 4g OD<br>Ibuprofen 1.2g OD<br>Codeine 240mg OD<br>Nicotine Patch 21mg OD	Left inferior frontal, orbito-frontal and frontal-opercular contusions.<br>CT Scan – Diffuse injury, NVP

TBIC	11	Ongoing	20	M	Mod/Sev	RTA	1-29 minutes	Sodium Chloride 2 tablets BD<br>Paracetamol 1g QDS	Tiny right occipital-temporal subdural haematoma. Small left temporal pole and bilateral subfrontal haemorrhagic contusions.<br>CT Scan – Diffuse injury
TBIC	11	Ongoing	48	M	Mod/Sev	Fall	1-29 minutes	Paracetamol 1g QDS<br>Ibuprofen 400mg TDS<br>Dihydrocodeine 60mg QDS<br>Phenytoin 300mg OD	Left frontal and middle cranial fossa subdural haematoma. Left inferior and middle temporal gyri haemorrhagic contusions.<br>CT Scan –a Diffuse injury
TBIC	11	Ongoing	30	M	Mod/Sev	RTA	Unknown	Paracetamol 1g PRN	Normal Scan<br>CT Scan – Diffuse injury
TBIC	12	1-24 hours	40	M	Mod/Sev	RTA	1-29 minutes	Paracetamol 1g QDS<br>Cyclizine 50mg TDS<br>Tramadol 50-100mg QDS	Small bilateral subfrontal contusions in the gyri recti. Left temporal lobe, superior temporal gyrus, frontal lobe and sensory cortex contusions.<br>CT Scan – Diffuse injury
TBIC	12	1-24 hours	27	F	Mod/Sev	Fall	<1 minute	Dihydrocodeine	Bilateral subfrontal haemorrhagic

								90mg<br>Paracetamol 4g<br>Co-Amoxiclav 975mg TDS	contusions. Thin right subdural collection. Minimal subarachnoid haemorrhage in the sulci over the right vertex.<br>CT Scan – Diffuse Injury
TBIC	12	< 1 minute	60	M	Mod/Sev	Fall	> 7 days	Dihydrocodeine 60 mg QDS<br>Oramorph 5mg OD<br>Amlodipine 10mg OD<br>Fluoxetine 20mg OD<br>Vitamin tablet I<br>Folic Acid 5mg OD<br>Detformin 850mg BD<br>Amitriptyline 75mg OD<br>Atorvastatin 20mg OD<br>Gliclazide 160mg BD	Frontal haematoma measuring more than 25ml. Smaller right temporal pole and left temporal lobe haemorrhagic contusion. Small middle cranial fossa and anterior cranial fossa subdural haematomas.<br>CT Scans – Mass lesions

								Bisoprolol 2.5mg<br>Irbesartan 300mg<br>Cyanocobalamin 50mg<br>Sitagliptin 100mg OD<br>Bendroflumethiazide 2.5mg	
TBIC	12	1-24 hours	36	M	Mod/Sev	Cycling	1-29 minutes	Dihydrocodeine 60mg QDS	Posterior right temporal lobe, occipital lobe, and temporal pole microhaemorrhages.<br>CT Scan – Diffuse injury, NVP
