# Supplementary material for: Disconnection between the default mode network and medial temporal lobes in post-traumatic amnesia
Source: Brain. 2016 Oct 22;139(12):3137–50. doi: 10.1093/brain/aww241 (PMC5382939; doi:10.1093/brain/aww241)
Supplement: Supplementary Data [file aww241_supp.zip › brain-2015-02273-File014.pdf]

	BASELINE			
**Analysis/Group**	**Healthy Controls**	**PTA Patients**	**TBI Controls**	**Total Patients**
**Neuropsychological Assessment**	17	11	8	19
**Resting-State Analysis**	15	8	7	15
**DTI Analysis**	16	6	6	12
	**FOLLOW-UP**			
	**Healthy Controls**	**PTA Patients**	**TBI Controls**	**Total Patients**
**Neuropsychological Assessment**	N/A	5	4	9
**Resting-State Analysis**	N/A	4	3	7
**DTI Analysis**	N/A	2	3	5
