# Supplementary material for: Disconnection between the default mode network and medial temporal lobes in post-traumatic amnesia
Source: Brain. 2016 Oct 22;139(12):3137–50. doi: 10.1093/brain/aww241 (PMC5382939; doi:10.1093/brain/aww241)
Supplement: Supplementary Data [file aww241_supp.zip › brain-2015-02273-File015.pdf]

	TASK									
	**PAL - Total Errors (6 shapes)**	**CRT - MCL (ms)**	**PRM - % Correct**	**PRM - MCL (ms)**	**SRM - % Correct**	**SRM - MCL (ms)**	**SWM - Between Errors**	**VRM FR – Total Correct**	**VRM IR - Total Correct**	**VRM DR – Total Correct**
**GROUP**	**BASELINE (mean +/- SD)**									
**Controls**	1.53 (1.59)	295.93 (39.22)	94.85 (4.78)	1898.70 (728.54)	80.88 (9.88)	1973.46 (784.92)	12.76 (11.94)	11.45 (3.91)	34.36 (1.80)	34.00 (2.10)
**PTA**	22.91 (15.20)	590.55 (295.43)	83.33 (11.49)	3219.24 (1077.30)	63.00 (31.54)	4578.68 (4363.02)	40.43 (24.84)	3.25 (1.71)	26.33 (5.86)	25.00 (3.46)
**TBI Controls**	1.63 (1.69)	402.15 (121.05)	88.02 (10.31)	2174.91 (705.91)	77.14 (11.85)	2828.64 (1262.46)	25.86 (15.10)	10.57 (10.98)	33.17 (2.32)	33.50 (2.26)
	**STATISTICAL TEST: ANOVA GROUP EFFECT**									
**F**	24.25	9.43	5.343	7.58	2.67	3.57	7.495	2.21	10.17	18.14
**p-value**	**p<0.001**	**p<0.001**	**p=0.011**	**p=0.002**	**p=0.09**	**p=0.042**	**p<0.002**	**p=0.138**	**p=0.0013**	**p<0.001**
	**FOLLOW-UP (mean +/-SD)**									
**Controls**	N/A	N/A	N/A	N/A	N/A	N/A	N/A	N/A	N/A	N/A
**PTA**	9.80	357.86	87.50	2306.84	83.33	2295.64	24.33	N/A	N/A	N/A

	(17.51)	(58.73)	(7.22)	(700.11)	(5.77)	(197.02)	(20.01)			
**TBI Controls**	2.50 (2.38)	320.73 (48.30)	95.83 (4.81)	1813.34 (203.81)	85.00 (4.08)	2076.20 (234.34)	12.00 (12.57)	N/A	N/A	N/A
	**STATISTICAL TEST: MIXED-EFFECTS MODEL**									
**Group**	F=8.38, **p=0.011**	F=2.74, **p=0.12**	F=3.61, **p=0.125**	F=6.48, **p=0.018**	F=0.70, **p=0.439**	F=2.24, **p=0.165**	F=0.34, **p=0.584**	N/A	N/A	N/A
**Timepoint**	F=3.3, **p=0.089**	F=5.1, **p=0.04**	F=0.02, **p=0.893**	F=0.55, **p=0.09**	F=4.95, **p=0.069**	F=1.60, **p=0.235**	F=4.53, **p=0.086**	N/A	N/A	N/A
**Group x Timepoint**	F=3.67, **p=0.076**	F=2.15, **p=0.166**	F=0.49, **p=0.535**	F=4.48, **p=0.071**	F=5.08, **p=0.433**	F=2.85, **p=0.126**	F=3.57, **p=0.132**	N/A	N/A	N/A
