# Supplementary material for: Disconnection between the default mode network and medial temporal lobes in post-traumatic amnesia
Source: Brain. 2016 Oct 22;139(12):3137–50. doi: 10.1093/brain/aww241 (PMC5382939; doi:10.1093/brain/aww241)
Supplement: Supplementary Data [file aww241_supp.zip › brain-2015-02273-File009.pdf]

Supplementary Material

Supplementary Methods

Paired Associates Learning (PAL) Task

The PAL task assesses object-location associative memory. The task consists of a number of levels, each of which has an encoding and a retrieval phase. At each level, boxes are shown on the screen. These boxes open up one at a time to reveal an object inside (encoding). After a short delay, objects appear in the middle of the screen, one at a time. The participant then responds to each stimulus by touching the box that they believe represents the original location of the object (retrieval). The version of the PAL task used includes eight levels (ie. memory for 1-8 objects was assessed). Performance on the PAL was based on level six, as not all patients were able to complete level eight. In addition, the number of errors at level six has been shown to be the measure particularly sensitive to memory dysfunction in Alzheimer's disease (Swainson, Hodges, *et al.* , 2001).

Structural and Functional Magnetic Resonance Imaging Acquisition

Standard clinical MR imaging was collected, including a T2-weighted FLAIR scan (26 5-mm-thick axial slices (1mm gap), TR = 8010 ms, TE = 150 ms, FA = 111°, matrix size = 512 x 256, field of view = 23 x 23 cm) and a T2* susceptibility-weighted (SWAN) scan (one 1.8-mm-thick axial slice, TE = 24.3 ms, FA = 15°, field of view = 20 x 20 cm) to assess microbleeds.

Functional T2*-weighted resting state data were collected using gradient echo EPI. A total of 210 image volumes of 36 slices (3.6 mm thickness, no interslice gap) were acquired in an interleaved manner per participant during a 7-minute scan (TE = 30 ms, TR = 2000ms, flip angle (FA) = 90°, in-plane resolution = 3.75 x 3.75mm, matrix

size = 64x64, field of view = 24x24 cm). Participants were asked to keep their eyes open during the resting state scan.

Structural MRI imaging was also collected, including a T1-weighted high-resolution FSPGR scan (156 1-mm-thick axial slices, TR = 8.516 s, TE = 3.336 ms, FA = 12°, in-plane resolution = 1x1mm, matrix size = 256x256, field of view = 25.6x25.6 cm) and diffusion tensor imaging (DTI). Diffusion-weighted volumes were acquired using a 30-direction protocol (52 slices, slice thickness = 2.3mm with a 0.2mm gap, field of view = 24x24cm, matrix size = 128x128, TR = 7000ms, b-value = $1000mm^2.s^{-1}$). One non diffusion-weighted image was also acquired (b-value = $0mm^2.s^{-1}$).

Lesion Analysis

Lesion locations were reported by a neuroradiologist (Supplementary Fig.1). Lesions were then manually defined as binary masks in the space of the native high-resolution T1, using the MRICron tool (http://www.mccauslandcenter.sc.edu/mricro/mricron/). Lesion masks were then registered to standard MNI152 1mm standard space using FMRIB's Linear Registration Tool (FLIRT; (Jenkinson, Bannister, *et al.* , 2002)). Registration parameters were defined by registering the participant's skull-stripped structural T1 image to the MNI standard template. Overlap images were then created using MRICron (Supplementary Fig.2). Lesion size was determined using fslstats and group differences were assessed with the use of a two-tailed Welch's Two-Sample t-test in R.

Functional MRI: Preprocessing

The first two volumes of the resting state data were removed to account for the T1 equilibrium effect. Resting state data pre-processing included a rigid-body realignment to correct for motion between volumes, followed by spatial smoothing using an 8 mm full-width at half-maximum Gaussian kernel. Functional images were registered to standard MNI space using FLIRT (Jenkinson, Bannister, *et al.*, 2002). Registration parameters were defined by registering the participant's skull-stripped

structural T1 image to the MNI standard template. Skull stripping was performed using the Brain Extraction Tool (BET) within FSL (Smith, 2002).

Data denoising included (1) temporal frequency filtering using a high-pass filter of 100 seconds (2) regression of the six motion parameters estimated from realignment and their first order temporal derivatives (twelve motion parameters in total) to remove any motion-correlated signal (Patel, Kundu, *et al.* , 2014) and (3) regression of cerebrospinal fluid (CSF) and white matter (WM) signal, extracted for each participant using the FAST (FMRIB's Automated Segmentation Tool; (Zhang, Brady, *et al.* , 2001)) segmented structural T1 images. CSF and WM segmentations were thresholded at 80% probability to ensure accurate tissue classification (Biswal, Mennes, *et al.* , 2010). To further investigate the effects of motion on the functional data a sub-analysis was performed, excluding a single patient who exceeded a relative root mean squared frame-wise displacement threshold of 0.3mm (see Supplementary Results).

Additional Brain Network Analyses – Functional Connectivity

We performed additional functional connectivity analyses on visual, fronto-parietal and executive control networks. PCC connectivity to visual areas and higher-order cognitive networks was investigated using targeted ROI analyses. The ROIs included the binarised visual, bilateral fronto-parietal and executive control networks, generated by the Smith et al., (2009) resting state data. To further investigate the role of other brain networks in PTA, group-level voxel-wise connectivity changes of these pre-defined networks were assessed. These included (1) a visual network thought to be unaffected by PTA and (2) fronto-parietal attentional and executive control networks, generated as above (Supplementary Fig.3A-C). The higher-order cognitive networks were used to investigate connectivity changes in brain areas thought not to be primarily involved in memory. In addition, areas identified as showing significant group-level voxel-wise alterations in FC at baseline, were used to determine whether connectivity normalized at follow-up. Linear mixed-effects models were used to assess these longitudinal effects. Group (PTA and TBI controls) and timepoint were defined as fixed effects, whereas subject was defined as a random effect to model

variability in subject intercepts. Post-hoc paired sample T-tests were used to investigate any significant main effects or interactions. The significant FC changes at baseline were correlated with neuropsychological data to investigate whether individual differences in connectivity were associated with the extent of cognitive impairment using Spearman's Correlation.

Structural MRI Connectivity: Diffusion Tensor Imaging

Diffusion data were corrected for motion artefacts and eddy currents, using affine transformations to register these images to the b = 0 image, prior to the creation of a brain mask generated by brain extracting the b = 0 image (using BET within FSL; Smith, 2002). A tensor model was then fitted to the data using FMRIB's Diffusion Toolbox (FDT) in FSL, constrained by the brain mask. Applying this tensor model generated voxel-wise individual subject fractional anisotropy (FA), mean diffusivity (MD), axial diffusivity (AD) and radial diffusivity (RD) maps. These were then warped into standard space and skeletonised using tract-based spatial statistics (TBSS) within FSL (Smith, Jenkinson, *et al.* , 2006), minimising the risk of partial volume effects.

Supplementary Results

Correspondence between Westmead PTA Scale and PAL Scores

There was no significant correlation was found between the Westmead and PAL scores (Spearman's rho = -0.31, p=0.204).

Neuropsychological Performance at Baseline

There was a significant group effect on the choice reaction time task, driven by significantly slower mean reaction times in the PTA group compared to both control groups (versus healthy - t(9.19)=-3.14, p=0.005; TBI controls -

t(12.74)=1.81,p=0.047), as well as slower response times in the TBI control group compared to healthy controls (t(6.52)=-2.17,p=0.03) (see Supplementary Table 3 for all ANOVA statistics). Spatial working memory performance also showed a group effect, driven by increased error rates in the PTA (t(7.17)=-2.81,p=0.013) and TBI control (t(9.27)=-2.05,p=0.035) groups compared to healthy controls. A significant group effect was also seen in pattern recognition memory accuracy and mean reaction times conditions, with the PTA group demonstrating a reduced percentage of correct responses compared to healthy controls (t(5.62)=2.38,p=0.029) and slower response times compared to both control groups (healthy-t(6.69)=-2.95,p=0.014; TBI-t(10.31)=2.33,p=0.035). Spatial recognition memory showed a group effect in terms of mean reaction times, driven by slower response times in the TBI control group compared to healthy controls (t(7.99)=-1.66,p=0.07). Spatial recognition accuracy was not significantly affected. Immediate and delayed verbal recognition memory showed significant group effects. Immediate recognition impairments showed a trend towards a lower number of correct responses in the PTA group compared to both control groups (healthy–t(2.10)=2.34,p=0.07; TBI–t(2.32)=-1.95,p=0.09). Delayed verbal recognition impairments were driven by a significantly lower number of correct responses in the PTA group in comparison to healthy (t(2.41)=4.29,p=0.018) and TBI controls (t(2.89)=-3.86,p=0.016). Free verbal recall was unaffected at a group level.

Changes in cognitive or imaging measures were not significantly affected by variability in follow-up length

Overall, changes in cognitive performance were not dependent on the variability in time between baseline and follow-up scans. The number of months in between scans did not correlate with the changes in associative memory (Spearman's rho=-0.28, p=0.47), information processing (Spearman's rho=0.13, p=0.75), pattern recognition (Spearman's rho=0.36, p=0.55) or spatial recognition memory (Spearman's rho=-0.45, p=0.37). There was a trend towards significance for spatial working memory (Spearman's rho=0.73, p=0.10).

In addition, variability in follow-up was not correlated with functional connectivity changes over time in parahippocampal (Spearman's rho=0.24, p=0.61) or precuneus/parietal (Spearman's rho=-0.07, p=0.88) areas.

There are no consistent areas of focal brain injury associated with PTA

We found no single area of focal brain injury present across all of the PTA group (Fig.2A-B). The maximal areas of lesion overlap were in the orbitofrontal cortex and temporal lobes for both the PTA group (N=4 max. overlap) and TBI controls (N=3 max. overlap). In the PTA group, the peak overlap was in the left inferoposterior temporal and right inferior frontal lobes. For the TBI controls, the peak overlap was in the left inferoanterior temporal and left inferior frontal lobes. This pattern of damage is generally typical for TBI (Gurdjian, 1975). In one patient of each group there was no evidence of focal lesions. There were no significant differences in lesion volume (mm^3) between the two groups (t(7.43)=1.29,p=0.24). Lesions ranged from 765-237,775 mm^3 in the PTA group and 25-66,730 mm^3 in the TBI controls.

Additional brain network analyses

To investigate whether the connectivity changes were specific to the DMN and MTL, we investigated FC within a number of other brain networks. The visual network was used as a control to test whether there were general changes in FC during PTA. No voxel-wise group differences were found in the PTA group. In addition, no significant group connectivity changes between the PCC and the same visual network were found.

We then assessed intrinsic connectivity networks involved in supporting cognitive function. Altered FC in all three higher-order cognitive networks was found. At a whole-brain voxel-wise level, directly comparing the PTA and healthy control groups showed significantly increased FC from the right fronto-parietal network to areas of the brain including the cingulo-opercular cortices, middle frontal gyrus, pre/post central gyri and precuneus (Supplementary Fig. 3A). The left fronto-parietal network also showed increases in FC in the PTA group to brain areas including the superior

frontal gyrus, paracingulate and cingulate cortices and the precuneus (Supplementary Fig.3B). Increased FC in the PTA group was also seen within the executive control network when compared to healthy controls, including in the anterior cingulate, bilateral insula, operculum and inferior frontal cortices (Supplementary Fig.3C). The executive control network also showed decreases in FC in the PTA group to the hippocampus and cerebellum (Supplementary Fig.3C). TBI controls showed no differences in FC with either healthy control or PTA groups.

PCC connectivity to these higher-order cognitive networks was also examined. PCC connectivity to the left fronto-parietal network showed a significant group effect ($F(2,27)=4.58$, $p=0.019$), driven by trend level increases in FC in both the PTA group ($t(7.61)=-2.05$, $p=0.08$) and TBI controls ($t(8.37)=1.43$, $p=0.099$) compared to healthy controls. PCC FC to the right fronto-parietal network showed a trend towards a group effect ($F(2,27)=3.28$, $p=0.053$), although post-hoc analyses were not significant. FC between the PCC and the executive control network showed a trend level group effect ($F(2,27)=2.91$, $p=0.072$), driven by decreased FC in the TBI control group in comparison to controls ($t(7.03)=1.99$, $p=0.086$). No differences were found between the healthy control and PTA groups.

Connectivity changes within the cognitive networks were not significantly correlated with either associative memory or choice reaction time measures. This was true across all subjects and in patients alone. Reaction time measures associated with the pattern recognition memory task were correlated with FC changes of the right fronto-parietal network and executive control network in the patient group. Specifically, increases in fronto-parietal connectivity to areas of the brain including the post-central and middle frontal gyrus were correlated with longer reaction times ($rho=0.81, p=0.0014$; $rho=0.80, p=0.002$). A similar pattern was observed in left inferior frontal areas of the executive control network ($rho=0.755, p=0.005$).

At follow-up, there was no significant change in fronto-parietal FC. In contrast, FC increases in left inferior frontal areas within the executive control network normalised at follow-up. A significant group by timepoint interaction ($F(1,5)=7.42$, $p=0.042$) appeared to be driven by decreased connectivity in the PTA group compared to

increases in the TBI control group over time, although post-hoc analyses were not significant (Supplementary Fig.3D).

Control Analyses – Motion

Motion across all participants was minimal (<0.5mm in relative root mean squared frame-wise displacement; RMSFD). PTA patients and TBI controls demonstrated an average relative RMSFD of 0.16 mm and 0.12 mm respectively, compared to 0.06 mm in healthy controls. These differences in mean displacement were of borderline significance when comparing PTA patients and healthy controls ($t(7.31)=-2.25$, $p=0.049$), and demonstrated a trend when comparing TBI and healthy control groups ($t(6.58)=-1.93$, $p=0.098$). There were no significant differences in movement between the patient groups ($t(11.94)=0.87$, $p=0.404$).

All participants except one demonstrated an RMSFD of under 0.3mm. Only one participant exceeded this value (0.43mm). To establish whether this participant was driving the connectivity results, an FC sub-analysis was performed excluding this patient and this showed qualitatively similar results. In addition, FC changes between the PCC and vmPFC ROI ($F(2,26)=3.49$, $p=0.045$) were still significant at the group level. The normalisation at follow-up of PCC FC with the PHIPP was not altered by this exclusion, as the outlier patient was not initially included in this analysis due to not having a follow-up timepoint.

Figure Legends

Supplementary Figure 1. Structural damage in all traumatic brain injury patients visible on T2 FLAIR images. Cont. = contusions. Damage in one patient is displayed on a T1 structural image due to a missing T2 FLAIR.

Supplementary Figure 2. Overlap map of lesions visible on T1 structural imaging for postr-traumatic amnesia (PTA) patients (A) and traumatic brain injury (TBI) controls (B). The colour of the map indicates the number of patients with a lesion in that area.

Supplementary Figure 3. Functional connectivity (FC) of the fronto-parietal and executive control networks. (A-C) The reference bilateral fronto-parietal and executive control resting state networks generated by Smith et al., (2009). (D-F) The direct contrast between PTA patients and healthy controls. Yellow-red colours indicate areas of increased FC in PTA patients compared to healthy controls. Blue areas indicate brain areas of reduced FC in PTA patients compared to healthy controls. Results are overlaid on the MNI152 T1 1mm brain template. (G) Graph represents connectivity changes within the executive control network at follow-up. All connectivity maps are significant at $p<0.05$, family-wise error (FWE) corrected. The heavy line within each boxplot indicates the sample median, with the range defined by the sample minimum and maximum.

Supplementary Table 1. Clinical characteristics and demographics of the traumatic brain injury patients. PTA = Post-traumatic amnesia; TBIC = TBI Controls; LOC = Loss of Consciousness; OD = omni die (once a day); BD = bis die (twice a day); TDS = ter die sumendus (three times a day); QDS = quarter die sumendus (four times a day); PRN = pro re nata (taken if needed); Mod/Sev = Moderate/Severe (Malec et al., 2007); RTA = Road Traffic Accident; CT = Computed Tomography; NVP = no visible pathology. PTA Duration is based on the IMPACT proforma (www.tbi-impact.org).

Supplementary Table 2. Breakdown of the numbers included in each analysis performed at both study time-points. PTA = post-traumatic amnesia, TBI = traumatic brain injury.

Supplementary Table 3. Neuropsychological performance in all experimental groups at both baseline and follow-up. PAL = paired associates learning, CRT = choice reaction time, PRM = pattern recognition memory, SRM = spatial recognition memory, SWM = spatial working memory, VRM FR = verbal recognition memory free recall, VRM IR = verbal recognition memory immediate recognition, VRM DR = verbal recognition memory delayed recognition, MCL = mean correct latency, PTA = post-traumatic amnesia, TBI = traumatic brain injury, ms = milliseconds, SD = standard deviation. PAL total errors (6 shapes) indicates the total number of errors

made at the 6-pattern stage, a measure that has been found to sensitively discriminate between controls and patients with Alzheimer's Disease (Swainson, Hodges, *et al.*, 2001) . SWM between errors represents the number of times a participant revisits a box in which a token has previously been found. This has been shown to be sensitive to impairments seen in Attention-Deficit Hyperactivity Disorder (Kempton, Vance, *et al.* , 1999).

References

Biswal BB, Mennes M, Zuo XN, Gohel S, Kelly C, Smith SM, *et al.* Toward discovery science of human brain function. Proc Natl Acad Sci U S A 2010;107:4734-4739.

Gurdjian ES. Re-evaluation of the biomechanics of blunt impact injury of the head. Surg Gynaecol Obstet 1975;140:845-850.

Jenkinson M, Bannister P, Brady M, Smith S. Improved Optimization for the Robust and Accurate Linear Registration and Motion Correction of Brain Images. NeuroImage 2002;17:825-841.

Kempton S, Vance A, Maruff P, Luk E, Costin J, Pantelis C. Executive function and attention deficit hyperactivity disorder- stimulant medication and better executive function performance in children. Psychological Medicine 1999;29:527-538.

Patel AX, Kundu P, Rubinov M, Jones PS, Vertes PE, Ersche KD, *et al.* A wavelet method for modeling and despiking motion artifacts from resting-state fMRI time series. Neuroimage 2014;95:287-304.

Smith SM. Fast robust automated brain extraction. Hum Brain Mapp 2002;17:143-155.

Smith SM, Jenkinson M, Johansen-Berg H, Rueckert D, Nichols TE, Mackay CE, *et al.* Tract-based spatial statistics: voxelwise analysis of multi-subject diffusion data. Neuroimage 2006;31:1487-1505.

Swainson R, Hodges JR, Galton CJ, Semple J, Michael A, Dunn BD, *et al.* Early Detection and Differential Diagnosis of Alzheimer's Disease and Depression with Neuropsychological Tasks. Dement Geriatr Cogn Disord 2001;12:265-280.

Zhang Y, Brady JM, Smith SM. Segmentation of Brain MR Images Through a Hidden Markov Random Field Model and the Expectation-Maximization Algorithm. IEEE Transactions on Medical Imaging 2001;20:45-57.
