# Supplementary material for: Disconnection between the default mode network and medial temporal lobes in post-traumatic amnesia
Source: Brain. 2016 Oct 22;139(12):3137–50. doi: 10.1093/brain/aww241 (PMC5382939; doi:10.1093/brain/aww241)
Supplement: Supplementary Data [file aww241_supp.zip › brain-2015-02273-File010.pdf]

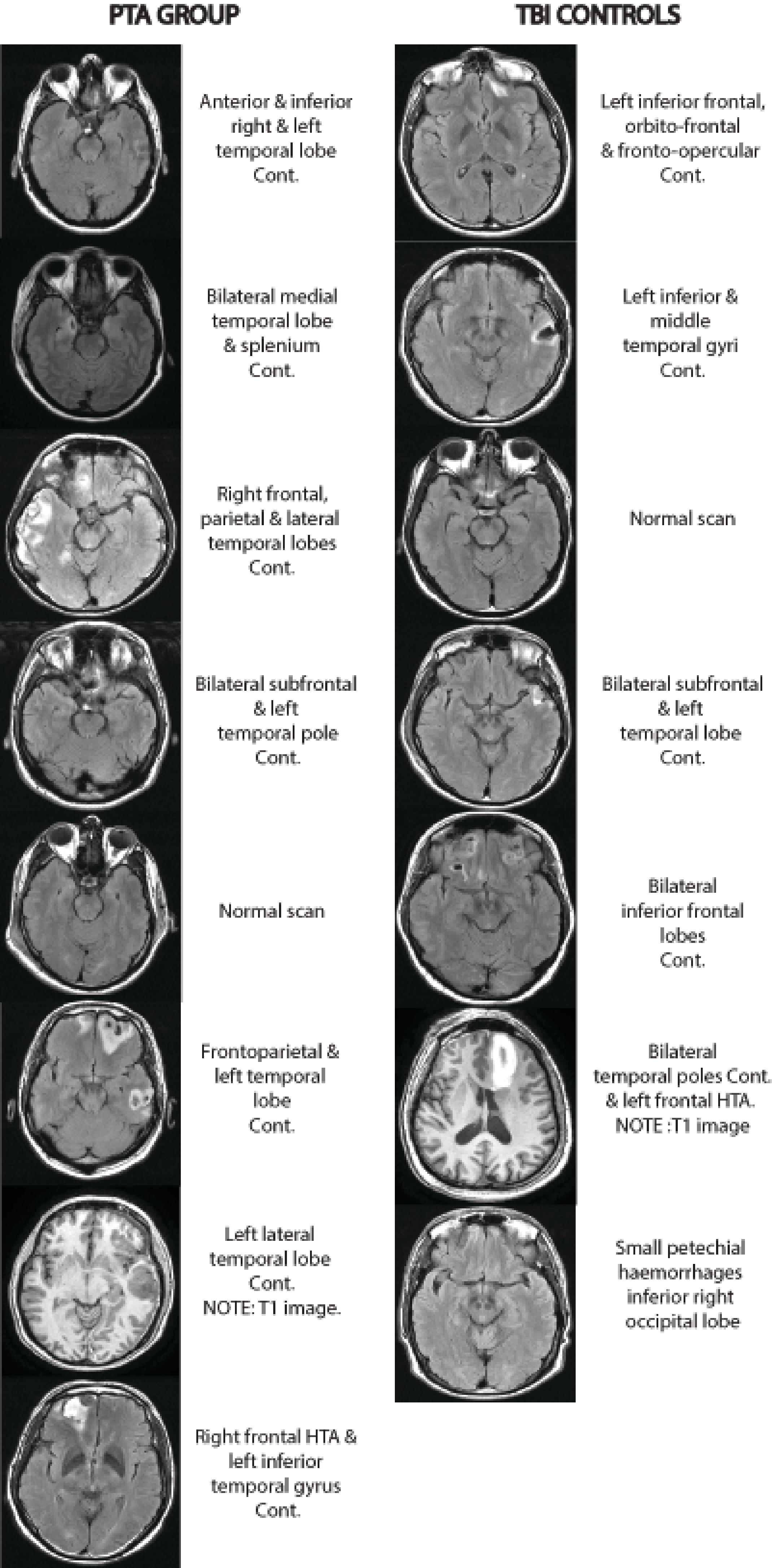
PTA GROUP
TBI CONTROLS
Anterior & inferior right & left temporal lobe Cont.
Left inferior frontal, orbito-frontal & fronto-opercular Cont.
Bilateral medial temporal lobe & splenium Cont.
Left inferior & middle temporal gyri Cont.
Right frontal, parietal & lateral temporal lobes Cont.
Normal scan
Bilateral subfrontal & left temporal pole Cont.
Bilateral subfrontal & left temporal lobe Cont.
Normal scan
Bilateral inferior frontal lobes Cont.
Frontoparietal & left temporal lobe Cont.
Bilateral temporal poles Cont. & left frontal HTA. NOTE :T1 image
Left lateral temporal lobe Cont. NOTE: T1 image.
Small petechial haemorrhages inferior right occipital lobe
Right frontal HTA & left inferior temporal gyrus Cont.
