# Supplementary figures and images for: Disconnection between the default mode network and medial temporal lobes in post-traumatic amnesia
Source: Brain. 2016 Oct 22;139(12):3137–50. doi: 10.1093/brain/aww241 (PMC5382939; doi:10.1093/brain/aww241)

**A** PTA GROUP

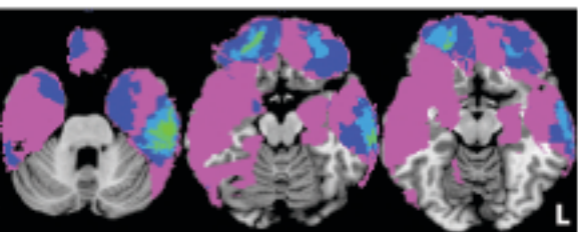

**B** TBI CONTROLS

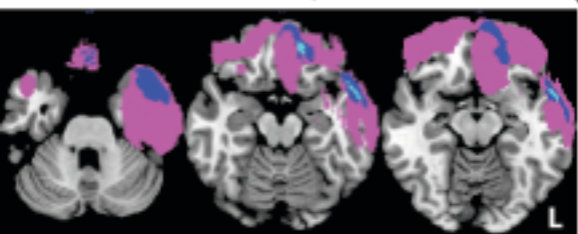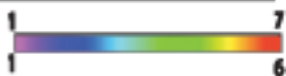

Supplement: Supplementary Data [file aww241_supp.zip › brain-2015-02273-File011.pdf]
